# Systematic Literature Review of the Prevalence, Pattern, and Determinant of Multimorbidity Among Older Adults in Nigeria

**DOI:** 10.1177/23333928231178774

**Published:** 2023-06-26

**Authors:** Abdulsalam Ahmed, Hafiz T.A. Khan, Muili Lawal

**Affiliations:** College of Nursing, Midwifery, and Healthcare, University of West London, London, United Kingdom of Great Britain and Northern Ireland

**Keywords:** multimorbidity, prevalence, pattern, determinant, older adults, Nigeria

## Abstract

**Introduction:**

Multimorbidity is a rising health issue globally and it is likely to become challenging in developing countries like Nigeria as they experience economic, demographic, and epidemiological transition. Yet, evidence of prevalence and patterns of multimorbidity, and their determinants, are scarce. This study aims to systematically review studies of the prevalence, patterns, and determinants of multimorbidity in Nigeria.

**Methods:**

Studies were identified by searching 5 electronic databases (PubMed, Web of Science, CINAHL, PsycINFO, Africa Index Medicus/Global Index Medicus). Multimorbidity as well as other versions of it was used to search. The prevalence and determinants were also searched. According to preestablished inclusion criteria, and using different search strategies, 6 articles were included. The quality and risk of bias were assessed using Joanna Briggs Institute appraisal tool for prevalence studies. Two researchers assessed the eligibility of studies for inclusion. The protocol was registered on PROSPERO Ref no. CRD42021273222. The overall prevalence, pattern, and determinants were analyzed.

**Results:**

We identified 6 eligible publications describing studies that included a total of 3332 (men 47.5%, women 52.5%) patients from 4 states plus the federal capital territory Abuja. The multimorbidity prevalence ranges from 27% to 74% among elderly Nigerians. Cardiovascular together with metabolic and/or musculoskeletal conditions were the frequent patterns of multimorbidity. A positive association was observed between age and multimorbidity in most studies. Other factors associated with multimorbidity were female gender, low education status, poor monthly income/unemployment, hospitalization, medical visits, and emergency services.

**Conclusion:**

There has been a growing need for more applied health services research to understand better and manage multimorbidity in developed countries. The scarcity of studies in our review reveals that multimorbidity is not a priority area of research in Nigeria, and this will continue to hinder policy development in that area.

## Introduction

The increase in average life span and transformation in global age structure toward a rapidly aging society is a major success for medical and public health systems. Although lengthening life spans is a global trend, the impact is not spread equally across the world.^1^ A huge difference still exists in life expectancy across the globe, it is said to be less than 60 years in sub-Saharan Africa and it is higher in first-world countries and even higher than 80 years in Japan.^2^ They also reported that global life expectancy increased from an average of 29 to 73 years in 2019. This success epitomizes new challenges for public health policy to ensure that healthy life expectancy is increased rather than just life expectancy.^3^ This is because an aging population presents many challenges and ignoring them could undermine the potential benefits and opportunities that living for longer can bring.^1^

Aging represents the greatest risk factor for disease and brings with it the chronic uncontrol of multiple organ systems.^4^ In other words, the uncompromising form of reality is that chronic diseases rarely occur in isolation, and as life expectancy increases, people acquire a growing number of illnesses.^5^ The number of people affected by multiple chronic diseases a condition termed multimorbidity is increasing dramatically around the world and caring for them has placed substantial stress on many health systems.^6^ Although this rising burden of chronic diseases has attracted the attention of public health researchers and policymakers worldwide, studies have shown that evidence on the epidemiology of multimorbidity in low- and middle-income countries (LMICs) is limited even though the region bears 80% of the global burden of noncommunicable diseases.^7^ Healthcare utilization and cost have surged in LMICs as a result of the prevalence of multimorbidity which places strain on the health system.^8,9^ Multimorbidity management requires a lot of resources that are hard work for both the patients and practitioners, especially when deepened with socioeconomic deprivation.^10^ Likewise, multimorbid patients are prone to frequent hospitalization, polypharmacy, treatment burden, and mortality.^11,12^

Recent studies reported that only 5% of multimorbidity research studies originated in LMICs, out of which were confined to only 6 middle-income countries (Brazil, China, South Africa, India, Mexico, and Iran).^13^ Similarly, most of the recognized studies on multimorbidity extrapolated from the global population through the largest systematic review of the prevalence of multimorbidity conducted up to date for over 25 years (1992-2017), by Nguyen et al^14^ were largely skewed to the other region of the world excluding Africa. This skewed distribution of multimorbidity studies demonstrates that there is a lack of attention on studying the phenomenon in other LMICs where it is likely to be more prevalent.

It has been estimated that the number of people experiencing multimorbidity is projected to rise along with population aging by >1% per annum until 2030.^15^ Therefore, there is a need for greater insight and an up-to-date understanding of the prevalence and patterns of multimorbidity, especially among the older population to inform preventive strategies in LMICs like Nigeria. There is a dearth of knowledge on the prevalence, pattern, and determinants of multimorbidity in Nigeria to our knowledge. And hence, this systematic review of the literature was conducted to determine (1) the prevalence of multimorbidity in older adults aged 60 years and above in Nigeria, (2) The common multimorbidity disease clusters in Nigeria, and (3) the determinant of multimorbidity disease in Nigeria.

## Methods

We conducted a systematic review that was preceded by a designed priori protocol (S1 File) following the Preferred Reporting Items for Systematic Reviews and Meta-Analyses (PRISMA) checklist (S2 File)^16^ and the PRISMA Protocols statement (S3 File),^17^ respectively. The Protocol was registered on PROSPERO Ref no. CRD42021273222 and available at https://www.crd.york.ac.uk/prospero/display_record.php?RecordID=273222.

## Inclusion and Exclusion Criteria

Included articles were peer-reviewed original articles with abstracts in English. However, conference presentations, opinion articles, books, and dissertations are excluded. The articles are observational cross-sectional articles on multimorbidity with a well-defined population 60 years and above conducted in Nigeria. The setting of the study was either community-based or health facility-based involving either or both inpatient and outpatient. Any other studies besides cross-sectional studies like cohort studies, and experimental studies were excluded. In addition, papers with single-review morbidity studies and without a clear description of the population were also excluded. Articles about the prevalence, pattern, and determinants of multimorbidity in Nigeria were included. Where multimorbidity has not been clearly defined, articles documenting 2 or more chronic medical conditions were considered in this (SR) even if they did not mention multimorbidity. Papers with single-review morbidity and studies with suboptimal methodology were excluded. No limitations were placed on the years of publication.

## Outcomes

Recent studies centered on defined multimorbidity as the simultaneous presence of more than one chronic medical condition in the same individual^18–22^ and multimorbidity patterns as the most frequent combination of specific disease pairs and the groups of health conditions with the highest degree of association using the corresponding statistical analyses of either cluster or factor analysis.^23^

## Search Strategy and Study Selection

We conducted an online literature search on PubMed, Web of Science, CINAHL, PsycINFO, and Africa Index Medicus/Global Index Medicus electronic databases, from inception up to August 16, 2021. Additionally, a corresponding internet search was done in Google Scholar, Google, and an online search from Africa Journal Online applying the same algorithm used in the bibliographic database search. However, where necessary the search was altered in line with the search engines and databases used. Screening of the reference list of included articles for the likelihood of relevant articles was conducted. The search terms included multimorbidity and other versions such as “multimorbidities,” “multi-morbidities,” “multi morbidity,” and multiple morbidities. We excluded “comorbidity” and other synonyms in our search strategy. And prevalence or epidemiology AND (pattern) AND determinants were used. This was done by using the “AND” and “OR” Boolean operators where appropriate (online supplement). These terms will be further restricted by location “Nigeria (Abia OR Adamawa OR Akwa Ibom OR Anambra or Bauchi or Bayelsa OR Benue OR Borno OR Cross River OR Delta OR Ebonyi OR Edo State OR Ekiti OR Enugu OR Gombe OR Imo OR Jigawa OR Kaduna OR Kano OR Katsina OR Kebbi OR Kogi OR Kwara OR Lagos OR Nasarawa OR Niger OR Ogun OR Ondo OR Osun OR Oyo OR Plateau OR Rivers OR Sokoto OR Taraba OR Yobe OR Zamfara OR ABUJA OR FCT).” The details of the search in specific databases are given in the study protocol (S1 File).

We downloaded and exported all identified citations to RefWorks referencing software manager. Duplicates were excluded using the Mendeley reference manager deduplication function. Afterward, the citations were exported from the reference manager into Rayyan systematic review software^24^ for the screening process. Title/abstract and full-text screening were carried out on the Rayyan software. The first reviewer (AA) screened the title and abstract of all initial articles. To ensure that appropriate studies were not unexploited a random 10% of all references were tested by the second reviewer (ML). AA and ML screened the full text independently, and any area of variance was fixed between the 2 reviewers and also with input from the third reviewer (HK). The most suitable data were included from multiple studies from the equivalent dataset. A PRISMA flow diagram is attached to show the detail of the study selection decisions made.

## Data Extraction

The extraction of data was conducted simultaneously with full-text searching. The relevant information was extracted from each article included and recorded immediately in the data extraction file (MS Excel). AA and ML independently extract the data. The extracted data are citation details: authors, title, journal, and year, details of the study: study setting (community or health facility) study design, period of data collection, location of the study, size of the sample, case definition, characteristics of the participants, description of main results: percentage prevalence of multimorbidity and the most common disease clusters in the study sample.

## Study Quality Assessment

Assessing the study characteristics and the risk of bias was done by 2 reviewers. The Joanna Briggs Institute (JBI) critical appraisal tool for prevalence studies was used.^25^ The JBI uses 9 items. There are 4 possible answers to the 9 items (yes, no, not clear, and not applicable). The threshold for the conversion of the JBI is as follows. Any item with a yes gets a score of 1, for no and unclear the score is zero, and for not applicable items are not included in the % calculation. A score of 60% and above was regarded as good quality. Based on the JBI data quality assessment system, all 6 studies were rated good quality. Four studies had a score of 100%, one study have 88%, and one study had 62.5%. The results from the 2 researchers were compared and differences were discussed between them.

## Ethics and Dissemination

This review received ethics approval as part of a larger project from the College of Nursing, Midwifery, and Healthcare, Research Ethics Panel (ethical approval number 1055). This research provided information on the prevalence of multimorbidity and other studied outcomes in Nigeria. The findings of this SR will be disseminated in a peer-reviewed journal article.

## Results

### Study Overview

**Figure 1.**
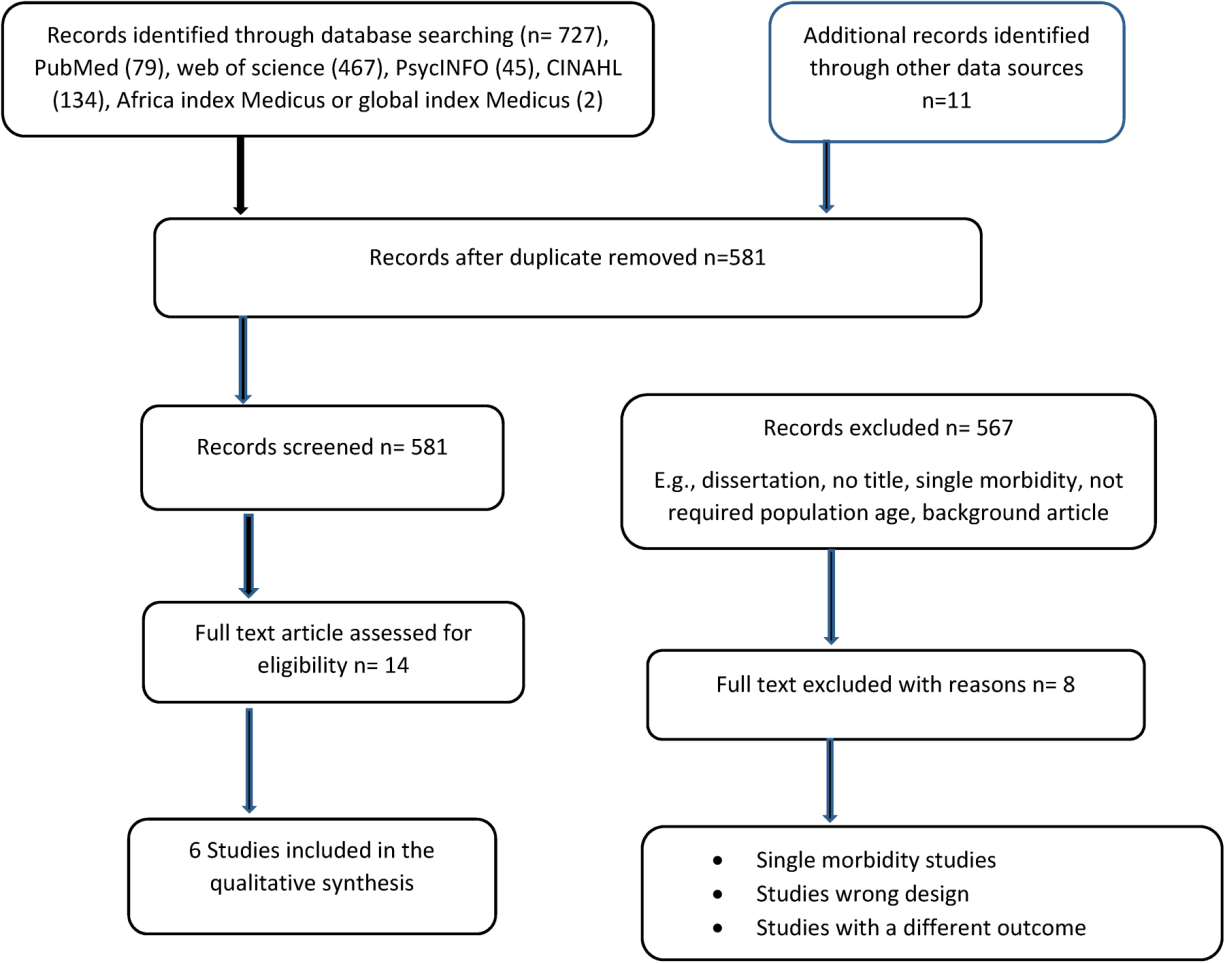
The PRISMA flowchart.

The processes of screening articles are shown on the PRISMA flowchart in Figure 1. The preliminary search yielded 738 titles; this was reduced to 581 after deduplication in the RefWorks citation manager software. In the title and abstract screening process, 567 were excluded leaving only 14 articles for assessment and only 6 were included in the narrative synthesis (S1 Table).

### Study Characteristics

Although the studies are relatively few, available ones are spread to cover the northern and southern parts of the country. Two studies each were conducted in north-central of Nigeria.^26,27^ Two studies were also conducted in Kano state northwestern Nigeria.^28,29^ One study each was conducted in the western and eastern parts of Nigeria, respectively.^30,31^ The total number of participants across the 6 studies was 3332 (men: 47.5%, women: 52.5%).^26–31^ The sample sizes of the included studies range from 333 to 1650 participants (see Table 1). All included studies were published after 2013, and the majority were in the last 6 years. Four studies conducted primary cross-sectional descriptive using either or a combination of clinical evaluation and administration of a questionnaire, interviews, and review of medical records.^26,28–30^ One study conducted a cross-sectional retrospective study over 12 months from January to December 2018,^27^ and the other study longitudinal prospective study both using medical health records.^31^ Two studies were carried out at the family medicine/outpatient department of Aminu Kano teaching hospital Kano, Nigeria, respectively.^28,29^ One study each was conducted at Nnamdi Azikiwe University Teaching Hospital, Nnewi Anambra state Nigeria,^31^ and General Out-Patient Clinic of the UATH Gwagwalada, Abuja, Nigeria,^27^ Osogbo, and Osun State, Nigeria, respectively.^30^ Four studies were hospital-based^27–29,31^ while 2 were conducted in the community.^26,30^

**Table 1.** Study Characteristics.

Study (state of study)	Study setting	Study design	Data collection period	Data source	Sample size	Age of participants
Nwani and Isah^31^ (Anambra state)	Nnamdi Azikiwe University Teaching Hospital (NAUTH), Nnewi Anambra State Nigeria	Prospective study	January 1, 2009, to December 31, 2009	Not reported	345 patients	Patients aged 65 years
Nicholas et al^27^ (Abuja)	General Out-Patient Clinic of the UATH Gwagwalada, Abuja.	Cross-sectional retrospective study	12 months, January 2018-December 2018	Medical health record	333 patients	60 and above years
Olawumi et al^28^ (Kano)	Conducted in the family medicine clinic (FMC) of Aminu kano teaching hospital kano	Descriptive cross-sectional study	October 5, 2020 to December 28 2020.	Clinical evaluation and administration of a questionnaire	348 patients	60 and above years
Abdulraheem et al^26^ (Niger)	The study was carried out in Niger State, Nigeria	A descriptive cross-sectional study	August 2014 to February 2015	Data were collected by questionnaire, interviews, review of medical records and clinical examination	Conducted among 1650 rural elderly populations attending primary healthcare centers	Age 60 years and above
Faronbi, Ajadi and Gobbens^30^ (Osun state)	Osogbo, Osun State, Nigeria.	A cross-sectional study	Data collection took 6 weeks (between October and November 2015)	Data were collected by questionnaire	400	60 years of age and above
Abdulazeez et al^29^ (Kano)	General Outpatient Clinic of Aminu Kano Teaching Hospital (AKTH) Kano	A descriptive cross-sectional study	May to June 2018.	Interviewer-administered questionnaire	384	60 years and above

### Study Quality Assessment

The result of the study quality assessment is provided in S2 Table. Of the included studies, 66% were high quality, and the remaining studies were medium quality. There was no study with low quality. The threshold for the conversion of the JBI is as follows. Any item with a yes gets a score of 1, for no and unclear the score is zero, and for not applicable items are not included in the % calculation. A score of 60% and above was regarded as good quality (high and medium). Based on the JBI data quality assessment system, all 6 studies were rated good quality. 4 studies had a score of 100%, 1 study has 88%, and 1 study had 62.5%. The results from the 2 researchers were compared and differences were discussed between them.

## Outcomes

### Primary Outcome

The definition of multimorbidity used, the number of disease conditions included in the study, and how the disease conditions were measured are all known facts that influence the prevalence of multimorbidity. However, all included studies used a “count” of the number of diseases to define multimorbidity, and multimorbidity was defined by having 2 or more diseases in an individual. All the included studies specified they were only focused on chronic conditions. One study qualified chronic diseases as compiled and counted by the World Health Organization (WHO).^31^ They stated that WHO defines chronic diseases as health problems that require ongoing management over a period of years or decades. Four studies draw and classify the chronic health problems of interest from the 147 International Classification of Primary Care, second edition (ICPC-2) rubrics list gathered by the Family Medicine Research Centre, University of Sydney.^26,28,29,31^ Two studies do not clearly measure.^27,30^

### Prevalence of Multimorbidity

All the included studies measured the prevalence of multimorbidity out of which 5 was among 60 years and above^26–30^ and 1 study uses 65 years and above.^31^ One study conducted in Oshogbo in Osun state reported the lowest prevalence of 27%^30^ while another conducted in Kano, kano state-reported a prevalence of 74%.^28^ The overall estimate ranges from 27% to 74.4% among elderly Nigerians. The most common study design observed is cross-sectional.^26–30^ In addition to the common cutoff point of 2 chronic diseases used in 5 studies, one study also investigated the prevalence estimate when multimorbidity was defined as “the co-occurrence of 3 or more chronic diseases.”^29^ The higher the number of diseases in the operational definition, the lower the prevalence (see Table 2).

**Table 2.** The Prevalence and Pattern of Multimorbidity Among Elderly Nigerians.

Study (state of study)	Prevalence (%)	Patterns of multimorbidity
Nwani and Isah^31^ (Anambra state)	The overall prevalence of multimorbidity among the elderly population studied was 49%. Two chronic diseases were present in 39.4% (n ¼ 136), whereas 3 or more chronic diseases were present in 9.6%.	No pattern was reported. However, a percentage of single morbidity was reported
Adams and Abubakar, 2018 (Abuja)	The majority 236 (71%) of the study participants had multiple morbidities.	No morbidity pattern was reported. The cardiovascular system was the most affected system with 227 of the study population followed by the musculoskeletal system 90 and 84 had metabolic derangement. The least affected system was the Ears Nose and Throat with 13 persons.
Olawumi et al^28^ (Kano)	The prevalence of multimorbidity in this study is 74.4%.	No multimorbidity pattern was reported. CV diseases were the most prevalent morbidity (88.5%) among the respondents, followed by the diseases of the musculoskeletal system (42%). Hypertension was the most prevalent (50%) among all the CV diseases.
Abdulraheem et al^26^ (Niger)	The percentage of participants with multimorbidity was 68.4% for 2 or more and 57.3 for 3 or more morbidities.	The most prevalent dyads of morbidities were hypertension and diabetes (31.4%), and hypertension and heart disease (25.6%). For triads of morbidities, the highest prevalence was found in HBP, diabetes and heart problem (10.3), and HBP, heart problem and osteoarthritis (9.8%).
Faronbi, Ajadi and Gobbens^30^ (Osun state)	This study also showed that multimorbidity is prevalent (27.0%) among the older adults in Nigeria.	No pattern was reported. However, percentage of single morbidity was reported
Abdulazeez et al^29^ (Kano)	More than half 190 (68.2%) of the participants had a 201(72.0%) had 2 or more chronic diseases (multimorbidity).	The commonest multimorbidity pattern based on system cluster was cardiometabolic–mechanical conditions 42 (15.1%). The top 3 frequent patterns of multimorbidity involving 2 clusters (dyad) of chronic diseases were hypertension–diabetes 7 (17%), followed by hypertension overweight 5 (12.1%) and hypertension osteoarthritis 4 (9.7%). The commonest triads of chronic diseases were hypertension–diabetes–osteoarthritis 15 (22.1%). The commonest quartet of chronic disease was hypertension diabetes–osteoarthritis–depression 3 (5.9%). The commonest quintet of chronic diseases was hypertension diabetes–osteoarthritis–visual impairment–obesity/overweight 3 (7.3%).

### Secondary Outcomes

#### Pattern of multimorbidity

The results of the studies were difficult to compare due to how data were reported. Only 2 studies reported the most frequent common pairs^26,29^ (see Table 2). The study by Abdulraheem et al^26^ reported the most prevalent dyads of morbidities were hypertension and diabetes (31.4%), and hypertension and heart disease (25.6%). For triads of morbidities, the highest prevalence was found in HBP, diabetes, and heart problem (10.3), and HBP, heart problems, and Osteoarthritis (9.8%).^26^ While Abdulazeez et al^29^ reported the commonest multimorbidity pattern based on system clusters was cardiometabolic–mechanical conditions at 15.1%.^29^ The top 3 frequent patterns of multimorbidity involving 2 clusters (dyad) of chronic disease were hypertension–diabetes (17%) followed by hypertension–overweight (12.1%) and hypertension–osteoarthritis (9.7). The commonest triad of chronic disease was hypertension–diabetes–osteoarthritis (22.1%). The commonest quartet of chronic diseases was hypertension–diabetes–osteoarthritis–depression (5.9%).^29^ The commonest quintet of chronic disease was hypertension–diabetes–osteoarthritis–visual impairment/overweight (7.3%). The remaining 4 studies were not structured to display the pairs but the pattern reported that hypertension was the commonest in all the studies.^29^

#### Determinant of multimorbidity

Age was the frequently studied determinant of multimorbidity.^5,32–35^ Although the determinant of multimorbidity was not assessed in all the included studies, 3 studies reported the determinants of multimorbidity.^26,27,29^ One study reported that multimorbidity occurred more in males (73.4% of males; OR = 1.062; CI = 0.926-1.219), aged 70 to 79 years (72.2%), unskilled workers (73.8%), and urban dwellers (73% of patients living in urban areas).^27^ Although *P* values show that these associations were not statistically significant however the relatively small sample size could account for this. However, a larger study by Abdulraheem et al^26^ showed that age and sex are independent risk factors for multimorbidity.^26^ They further stated that apart from age, factors most strongly and independently associated with multimorbidity were female gender, low education status, poor monthly income/unemployment, hospitalization, medical visits, and emergency services. One study reported that apart from age, factors most strongly and independently associated with multimorbidity were female gender, low education status, poor monthly income/unemployment, hospitalization, medical visits, and emergency services.^26^

Another study reported that participants with formal education were more than 30% less likely to have multimorbidity than those without formal education.^29^ Similarly, participants that were employed were almost 40% less likely to have multimorbidity than those that were unemployed.^29^ The same study reported that participants that were overweight/obese had higher chances of developing multimorbidity when compared with individuals with normal BMI. And that participants were functionally dependent were 20 times more likely to have multimorbidity than functionally independent elderly participants.^29^

## Discussion

This systematic review provides a well-informed and comprehensive analysis of multimorbidity prevalence, pattern, and determinants in Nigeria. We identified 6 articles across 5 states in Nigeria. Earlier authors of systematic literature reviews of multimorbidity also noted the limited representation of developing countries in multimorbidity research.^36^ In essence, multimorbidity is not a priority area of research in Nigeria, and this will continue to hinder policy development in that area. Our review shows that prevalence estimates varied markedly according to age, gender, marital status, marital setting, tribe, educational levels, adequate income, living conditions, family support, and operational definitions of multimorbidity.^26,27,29,31^ However, the findings from our studies were consistent with other previous studies and systematic reviews.^14,23,37^ Although the prevalence estimates varied between and within age groups, most studies in our sample indicated multimorbidity as a common phenomenon in individuals 60 years and above. Where prevalence estimates by gender were reported, it showed variation, 2 studies reported higher prevalence among men.^27,31^ However, in one study females appeared to have a higher multimorbidity prevalence than males in studies.^26^ This is suggestive of an association between sex and multimorbidity, evidence of which was provided in multiple studies.^14,38,39^ The higher the number of diseases in the operational definition, the lower the prevalence. This was observed in our review, the percentage of participants with multimorbidity was 68.4% for 2 or more and 57.3 for 3 or more morbidities.^26^ This finding supported an observation where it was found that from 44% when multimorbidity was defined as 2 diseases, the prevalence reduced to 27% for 3 diseases, 15% for 4 diseases, 7% for 5 diseases, and only 3% for 6 diseases.^40^ The highest prevalence estimates in our sample were reported in studies that used 2 diseases to define multimorbidity 71%^27^ and 74%.^28^ The combination of diseases may make multimorbidity prevalence differ significantly.^40,41^ In the existing literature, a range of different combinations has been proposed from a list of 16 chronic diseases^42^ to a list of 291 diseases^43^ and anything in between.^44^ Ferrer et al^42^ argued that an open list of diagnoses should be used since it gave the highest prevalence estimate. For our studies, most of the chronic health problems of interest were drawn from the 147 ICPC-2 rubrics list gathered by the Family Medicine Research Centre, the University of Sydney.^45^ The known fact is that there were no specific criteria for disease inclusion in these studies because of the lack of a standardized list, and they were often determined by the author's expertise and experience. However, the most common conditions included were those that have the highest prevalence of clinical relevance.

Although hypertension and diabetes mellitus appear to be common findings in this study, this is not completely different from other parts of the world. For example, in Korean adult men, diabetes mellitus and hypertension yielded the highest probability of multimorbidity, and for women polyarthritis and hypertension.^46^ In the United States, the most prevalent dyads of multimorbidity in men after 65 years of age is arthritis and hypertension followed by diabetes mellitus and hypertension in both sexes.^47^ The most prevalent cluster in all 4 strata of multimorbidity included hypertension diseases and metabolic disorders.^48^

## Strengths and Limitations

Our study selection, screening processes, search strategy, inclusion criteria, and quality assessment were wide-ranging and exhaustive. Our review included studies in both the hospital and communities and the first of its kind to conduct SLR of prevalence, pattern, and determinant of multimorbidity in Nigeria. This review, however, was not without limitations. Evaluations of prevalence, determinants, and patterns in our study are limited by the methods used in the primary studies. Similarly, all the measures of multimorbidity used in these studies were mostly disease count, and disease count is only one of the 20 measures to date.

## Conclusion

The overall prevalence estimates range from 27% to 74.4% among elderly Nigerians. However, the disease pair varies among studies, hypertension, and diabetes appear to be the commonest dyads. Determinants of multimorbidity in our study include age, gender, and lower socioeconomic status. The scarcity of studies in our review reveals that multimorbidity is not a priority area of research in Nigeria, and this will continue to hinder policy development in that area.

## Supplemental Material

sj-docx-1-hme-10.1177_23333928231178774 - Supplemental material for Systematic Literature Review of the Prevalence, Pattern, and Determinant of Multimorbidity Among Older Adults in Nigeria Click here for additional data file. Supplemental material, sj-docx-1-hme-10.1177_23333928231178774 for Systematic Literature Review of the Prevalence, Pattern, and Determinant of Multimorbidity Among Older Adults in Nigeria by Abdulsalam Ahmed, Hafiz T.A. Khan and Muili Lawal in Health Services Research and Managerial Epidemiology

sj-docx-2-hme-10.1177_23333928231178774 - Supplemental material for Systematic Literature Review of the Prevalence, Pattern, and Determinant of Multimorbidity Among Older Adults in Nigeria Click here for additional data file. Supplemental material, sj-docx-2-hme-10.1177_23333928231178774 for Systematic Literature Review of the Prevalence, Pattern, and Determinant of Multimorbidity Among Older Adults in Nigeria by Abdulsalam Ahmed, Hafiz T.A. Khan and Muili Lawal in Health Services Research and Managerial Epidemiology

sj-docx-3-hme-10.1177_23333928231178774 - Supplemental material for Systematic Literature Review of the Prevalence, Pattern, and Determinant of Multimorbidity Among Older Adults in Nigeria Click here for additional data file. Supplemental material, sj-docx-3-hme-10.1177_23333928231178774 for Systematic Literature Review of the Prevalence, Pattern, and Determinant of Multimorbidity Among Older Adults in Nigeria by Abdulsalam Ahmed, Hafiz T.A. Khan and Muili Lawal in Health Services Research and Managerial Epidemiology

sj-docx-4-hme-10.1177_23333928231178774 - Supplemental material for Systematic Literature Review of the Prevalence, Pattern, and Determinant of Multimorbidity Among Older Adults in Nigeria Click here for additional data file. Supplemental material, sj-docx-4-hme-10.1177_23333928231178774 for Systematic Literature Review of the Prevalence, Pattern, and Determinant of Multimorbidity Among Older Adults in Nigeria by Abdulsalam Ahmed, Hafiz T.A. Khan and Muili Lawal in Health Services Research and Managerial Epidemiology

sj-docx-5-hme-10.1177_23333928231178774 - Supplemental material for Systematic Literature Review of the Prevalence, Pattern, and Determinant of Multimorbidity Among Older Adults in Nigeria Click here for additional data file. Supplemental material, sj-docx-5-hme-10.1177_23333928231178774 for Systematic Literature Review of the Prevalence, Pattern, and Determinant of Multimorbidity Among Older Adults in Nigeria by Abdulsalam Ahmed, Hafiz T.A. Khan and Muili Lawal in Health Services Research and Managerial Epidemiology
