# Supplementary material for: Systematic Literature Review of the Prevalence, Pattern, and Determinant of Multimorbidity Among Older Adults in Nigeria
Source: Health Serv Res Manag Epidemiol. 2023 Jun 26;10:23333928231178774. doi: 10.1177/23333928231178774 (PMC10331101; doi:10.1177/23333928231178774)
Supplement: sj-docx-1-hme-10.1177_23333928231178774 - Supplemental material for Systematic Literature Review of the Prevalence, Pattern, and Determinant of Multimorbidity Among Older Adults in Nigeria [file sj-docx-1-hme-10.1177_23333928231178774.docx]

**S1 File: Study protocol**

**Title: Protocol for a systematic review of the prevalence, pattern, and determinant of multimorbidity in Nigeria**

**Registration:** Registered in PROSPERO (CRD42021273222).

**Authors: Abdulsalam Ahmed ^1^, Muilli Lawal ^1^, Hafiz khan ^1, *^**

1. College of Nursing, Midwifery, and Healthcare University of West London; yatalaa@gmail.com (A.A), Muililawal@uwl.ac.uk (M.L), Hafiz.Khan@uwl.ac.uk (H.K)

* Correspondence: Hafiz.Khan@uwl.ac.uk

**Authors’ contributions:** Conception of research idea (HK), Literature review (AA), Research protocol design (AA), Study appraisal (HK, ML and AA), Data extraction (ML and AA), Data analysis and interpretation of results (HK, ML and AA), Manuscript drafting (AA), and review of the initial and final draft of the manuscript (HK and ML)

**Source of Support:** University of West London

**Sponsor:** University of West London

**Role of sponsor:** The sponsor did not play any role in the development of the study protocol.

**Introduction**

The uncompromising form of reality is that chronic diseases rarely occur in isolation, above all as life expectancy increases, people acquire a growing number of illnesses (Barnett *et al.,* 2012). The number of people affected by multiple chronic diseases a condition termed multimorbidity is increasing dramatically around the world and caring for them has placed substantial stress on many health systems (Navickas *et al.,* 2016). Although the rising burden of chronic diseases has attracted the attention of public health researchers and policymakers worldwide, research has shown that evidence on the epidemiology of multimorbidity in Low- and Middle-Income Countries (LMICs) is limited even though the region bears 80% of the global burden of non-communicable diseases (NCDs) (Hunter and Reddy, 2013). Healthcare utilization and cost have surged in LMICs as a result of the prevalence of multimorbidity which places strain on the health system (Frølich *et al.,* 2019; Sum *et al.,* 2019) Studies have shown that multimorbidity management requires a lot of resources that are hard work for both the patients and practitioners, especially when deepened with socioeconomic deprivation (O'Brien *et al.,* 2011). Additionally, multimorbid patients are prone to frequent hospitalization, polypharmacy, and treatment burden, and mortality (Duerden, Avery and Payne, 2013; Palmer *et al.,* 2018).

Although, the rising burden of chronic diseases has attracted the attention of public health researchers and policymakers worldwide. Recent studies reported that only 5% of multimorbidity research studies originated in LMICs (Xu, Mishra and Jones, 2017). They further stated that most of (n ¼ 52, 68.4%) the available studies in LMICs were confined to only six middle-income countries (Brazil, China, South Africa, India, Mexico, and Iran). This skewed distribution of multimorbidity studies demonstrates that there is a lack of attention on studying the phenomenon in other LMICs where it is likely to be more prevalent. Similarly, most of the recognized studies on multimorbidity extrapolated from the global population through the largest systematic review of the prevalence of multimorbidity conducted up to date for over 25 years (1992-2017), by Nguyen et al. (2019) were largely skewed to the other region of the world excluding Africa.

It has been estimated that the number of people experiencing multimorbidity is projected to rise along with population ageing by >1% per annum until 2030 (Yoon *et al.,* 2014). Therefore, there is a need for greater insight and an up-to-date understanding of the patterns of multimorbidity, especially among the older population to inform preventive strategies in LMICs like Nigeria. Since data on prevalence and pattern of combination of multimorbidity is less documented in these regions

To our knowledge, no study has been done to assess the prevalence of multimorbidity in Nigeria. The proposed systematic review aims to synthesise existing literature on the prevalence of multimorbidity in Nigeria and identify common disease clusters and trends in the country.

**Research questions**

1. What is the prevalence of multimorbidity in older adults age 60 years and above in Nigeria?
2. What is the prevalence of multimorbidity in adult males and females stratified by age group in Nigeria?
3. What are common multimorbidity disease clusters in Nigeria?
4. What are the determinants of multimorbidity in Nigeria?

**Methods**

**Protocol and registration**

The methods for this systematic review were developed following the Preferred Reporting Items for Systematic Reviews and Meta-Analyses (PRISMA) checklist (or guidelines) (Moher, 2009) and the PRISMA Protocols statement (Shamseer *et al.,* 2015). This systematic review (SR) will be registered with PROSPERO, an ~~i~~nternational ~~p~~rospective database for the registration of systematic reviews.

**Inclusion criteria**

- **Study location**: studies conducted in any of the 36 states plus the federal capital territory in Nigeria.
- **Population/types of participants:** This systematic review will be restricted to people who reside in Nigeria and are 60 years and above. Ideally, articles should report on the prevalence of multimorbidity by age group and sex
- **Exposure:** Articles about the prevalence of multimorbidity in Nigeria will be included. For articles where multimorbidity has not been clearly defined, this (SR) will adopt an operational definition of multimorbidity to include the article with operational definition studies documenting two or more chronic conditions”, even though not mentioning the term multimorbidity.
- **Context:** Articles must report on studies conducted in Nigeria. The setting of the study can be community-based, or health facility-based involving either or both inpatient and outpatient.
- **Outcome:** The primary outcome will be the prevalence of multimorbidity in Nigeria.
- The secondary outcome will be disease clusters and determinants
- **Timeframe:** no limitations placed on the year of publication.
- **Language**: studies published in English only will be included.

**Exclusion criteria**

- Any other studies besides cross-sectional studies like longitudinal, cohort studies
- Exclude studies conducted in non-African countries
- Papers with single review morbidity
- Studies with suboptimal methodology
- Studies without a clear description of the population
- Case report, review articles, conference papers, unpublish reports, dissertations, abstract only paper

**Table 1 Summary of inclusion and exclusion criteria**

|  | **Inclusion criteria** | **Exclusion criteria** |
| --- | --- | --- |
| 1 | Peer-reviewed articles. | Non-peer-reviewed articles and grey literature |
| 2 | Observational cross-sectional studies. | Any other studies besides cross-sectional studies like longitudinal, cohort studies, Experimental studies. |
| 3 | Only studies conducted in Nigeria | Exclude studies conducted outside Nigeria |
| 4 | Articles about multimorbidity | Papers with single review morbidity |
| 5 | Studies with well define population 60 years and above | Studies without a clear description of the population |
| 6. | Only studies conducted in English will be conducted | Studies not published in English |

**Search strategy**

The search strategy for this (SR), will embrace the assessment of all relevant literature citations captured through the application of the search algorithm in five electronic bibliographic databases. Also, a literature search will be conducted via hand-searching references of selected (review) articles and conference proceedings. Additionally, a corresponding internet search will be done in Google Scholar, Google, and an online search from Africa Journal Online (AJOL) applying the same algorithm used in the bibliographic database search. However, the search strategy will be modified, where necessary, according to the database or search engine used. Reference lists of included articles will also be screened for relevant articles.

**Search term**

The search terms will include ‘multimorbidity’ and linguistic variations such as ‘multi-morbidity’, ‘multimorbidities’, ‘multi-morbidities’, ‘multi morbidity’, ‘multi morbidities’, multiple morbidities’, ‘multiple-morbidities’. Also included in the list are terms such as ‘multiple conditions’, ‘multiple diseases’, ‘multiple chronic diseases’, ‘multiple chronic conditions’, ‘multiple illnesses’, ‘multiple diagnoses’, ‘multipathology’, ‘chronic condition’, ‘chronic diseases. These terms will be further restricted by location ‘Nigeria (Abia OR Adamawa OR Akwa Ibom OR Anambra or Bauchi or Bayelsa OR Benue OR Borno OR Cross River OR Delta OR Ebonyi OR Edo State OR Ekiti OR Enugu OR Gombe OR Imo OR Jigawa OR Kaduna OR Kano OR Katsina OR Kebbi OR Kogi OR Kwara OR Lagos OR Nasarawa OR Niger OR Ogun OR Ondo OR Osun OR Oyo OR Plateau OR Rivers OR Sokoto OR Taraba OR Yobe OR Zamfara OR ABUJA OR FCT) and by method and study design ‘prevalence, epidemiology, pattern’. This will be done by using the ‘AND’ and ‘OR’ Boolean operators where appropriate.

**Library/Database: PubMed**

**Date of 16/08/2021**

Multimorbidity OR multi-morbidity OR multimorbidities OR multi-morbidities OR multi morbidities OR multiple morbidities OR multiple-morbidities OR multiple conditions OR multiple diseases OR multiple chronic diseases OR multiple chronic conditions OR multiple illnesses OR multiple diagnoses OR multipathology OR chronic condition OR chronic diseases AND Nigeria (Abia OR Adamawa OR Akwa Ibom OR Anambra or Bauchi or Bayelsa OR Benue OR Borno OR Cross River OR Delta OR Ebonyi OR Edo State OR Ekiti OR Enugu OR Gombe OR Imo OR Jigawa OR Kaduna OR Kano OR Katsina OR Kebbi OR Kogi OR Kwara OR Lagos OR Nasarawa OR Niger OR Ogun OR Ondo OR Osun OR Oyo OR Plateau OR Rivers OR Sokoto OR Taraba OR Yobe OR Zamfara OR ABUJA OR FCT) AND prevalence AND (pattern) AND determinants.

**Hits 79**

**Library/Database: Web of science**

**Date of 16/08/2021**

Multimorbidity OR multi-morbidity OR multimorbidities OR multi-morbidities OR multi morbidities OR multiple morbidities OR multiple-morbidities OR multiple conditions OR multiple diseases OR multiple chronic diseases OR multiple chronic conditions OR multiple illnesses OR multiple diagnoses OR multipathology OR chronic condition OR chronic diseases AND Nigeria (Abia OR Adamawa OR Akwa Ibom OR Anambra or Bauchi or Bayelsa OR Benue OR Borno OR Cross River OR Delta OR Ebonyi OR Edo State OR Ekiti OR Enugu OR Gombe OR Imo OR Jigawa OR Kaduna OR Kano OR Katsina OR Kebbi OR Kogi OR Kwara OR Lagos OR Nasarawa OR Niger OR Ogun OR Ondo OR Osun OR Oyo OR Plateau OR Rivers OR Sokoto OR Taraba OR Yobe OR Zamfara OR ABUJA OR FCT) AND prevalence AND (pattern) AND determinants.

**Hits 467**

**Library/Database: PsycINFO database**

**Date of 16/08/2021**

Multimorbidity OR multi-morbidity OR multimorbidities OR multi-morbidities OR multi morbidities OR multiple morbidities OR multiple-morbidities OR multiple conditions OR multiple diseases OR multiple chronic diseases OR multiple chronic conditions OR multiple illnesses OR multiple diagnoses OR multipathology OR chronic condition OR chronic diseases AND Nigeria AND prevalence or incidence or epidemiology or frequency or occurrence AND (pattern) AND determinants or factors or causes

**Hits 45**

**Library/database: CINAHL**

**Date of 16/08/2021**

Multimorbidity OR multi-morbidity OR multimorbidities OR multi-morbidities OR multi morbidities OR multiple morbidities OR multiple-morbidities OR multiple conditions OR multiple diseases OR multiple chronic diseases OR multiple chronic conditions OR multiple illnesses OR multiple diagnoses OR multipathology OR chronic condition OR chronic diseases AND Nigeria AND prevalence or incidence or epidemiology or frequency or occurrence AND (pattern) AND determinants or factors or causes

**Hits 134**

**Library/database: Africa Index Medicus or Global Index Medicus**

**Date of 16/08/2021**

Multimorbidity OR multi-morbidity OR multimorbidities OR multi-morbidities OR multi morbidities OR multiple morbidities OR multiple-morbidities OR multiple conditions OR multiple diseases OR multiple chronic diseases OR multiple chronic conditions OR multiple illnesses OR multiple diagnoses OR multipathology OR chronic condition OR chronic diseases AND Nigeria AND prevalence or incidence or epidemiology or frequency or occurrence AND (pattern) AND determinants or factors or causes.

**Hits 2**

| s/no | Database | Number of hits | Date of search |
| --- | --- | --- | --- |
| 1 | PubMed | 79 | 16/08/2021 |
| 2 | Web of science | 467 | 16/08/2021 |
| 3 | CINAHL | 134 | 16/08/2021 |
| 4 | PsycINFO | 45 | 16/08/2021 |
| 5 | Africa Index Medicus or Global Index Medicus | 2 | 16/08/2021 |
|  | **Total** | 727 |  |

**Data management**

Two reviewers will conduct the literature search and harmonised it afterward. Once the search is run, citations will be downloaded into an excel sheet and exported into RefWorks the referencing software manager. Duplicates will be excluded using the reference manager deduplication function. Afterward, the citations will be exported from the reference manager into Rayyan (Ouzzani *et al.,* 2016).

**Selection process**

The study selection will be conducted by two independent reviewers and a third reviewer will decide about uncertainties. In essence, the full text of potentially relevant articles will be retrieved and independently reviewed by two review authors for eligibility. A PRISMA flow diagram will detail the study selection decisions made. In the case of multiple studies from the same dataset, the most appropriate data will be included. Where additional information is needed, the authors of the study will be contacted. With not more than 2 attempts in a week, one reviewer will email the corresponding author.

**Data extraction process**

Extraction of data will be conducted simultaneously with full-text searching. The relevant information will be extracted from each article included and recorded immediately in the data extraction file (MS Excel). This will be carried out by two independent reviewers and two others will check the information. The following data will be extracted:

- Citation details: authors, title, journal, and year.
- Details of the study: study setting (community or health facility) study design, period of data collection, location of the study, and sample size.
- Case definition: how multimorbidity was defined and how disease conditions were measured.
- Characteristics of the participants: age, sex, urban/rural, socioeconomic characteristics, and the rest.
- Description of main results: percentage prevalence of multimorbidity (n/N) and 95% CIs. Prevalence of conditions stratified by age and sex. Information on the most common disease clusters in the study sample.
- In addition, the aims of the study, the method of data analysis used, and any points of difference that may affect the interpretation of findings will be noted

**Risk of Bias (quality) assessment**

Each article to be included for analysis will be a relevant article that meets the inclusion and exclusion criteria. The quality of each article that meets the inclusion and exclusion criteria will be independently evaluated. Assessing the study characteristics and the risk of bias by two reviewers. The Joanna Briggs Institute (JBI) critical appraisal tool for prevalence studies was used (Joanna Briggs Institute, 2017). The results from the two researchers will be compared and differences will be discussed between them. Where consensus is not reached, a third reviewer will be called on to intercede. Studies with a high risk of bias will be excluded and the reasons for their exclusion will be noted.

**Data synthesis**

The results will be presented per the PRISMA statement. Moderate to low-risk bias studies will be included in the analysis. For more clarity, information from the articles will be demonstrated graphically where necessary. Point and interval estimates will be summarized for each study. If information is available from the articles the prevalence data will be broken down by age, sex and disease clusters, and trends. Differences and similarities will be documented. Where sufficient data exist, subgroup analysis will be conducted based on multimorbidity case definition used, study setting (eg, community or health facility), age groups, sex, and periods. The findings from included articles will be summarized in tables which will include the year of data collection, the study type and setting (community or health facility-based), geographical location of the study, how multimorbidity is defined in each study, the diseases and number of diseases included in the study and how they were ascertained (e.g., measured, or self-reported).

**Ethics and dissemination**

Only published journal articles were included in the systematic review (This review received ethics approval as part of a larger project by the College of Nursing, Midwifery, and Healthcare research ethics panel of the University of the West London ethical committee). This research provided information on the prevalence of multimorbidity and other studied outcomes in Nigeria. Thus, contributing to the design of future research projects in this area. The findings of this SR will be disseminated in a peer-reviewed journal article.

**References**

Barnett, K., Mercer, S.W., Norbury, M., Watt, G., Wyke, S. and Guthrie, B. (2012) 'Epidemiology of multimorbidity and implications for health care, research, and medical education: a cross-sectional study', *The Lancet,* 380(9836), pp. 37-43.

Duerden, M., Avery, T. and Payne, R. (2013) 'Polypharmacy and medicines optimisation', *Making it safe and sound.London: The King’s Fund,* .

Frølich, A., Ghith, N., Schiøtz, M., Jacobsen, R. and Stockmarr, A. (2019) 'Multimorbidity, healthcare utilization and socioeconomic status: a register-based study in Denmark', *PloS one,* 14(8), pp. e0214183.

Hoy, D., Brooks, P., Woolf, A., Blyth, F., March, L., Bain, C., Baker, P., Smith, E. and Buchbinder, R. (2012) 'Assessing risk of bias in prevalence studies: modification of an existing tool and evidence of interrater agreement', *Journal of clinical epidemiology,* 65(9), pp. 934-939.

Hunter, D.J. and Reddy, K.S. (2013) 'Noncommunicable diseases', *New England Journal of Medicine,* 369(14), pp. 1336-1343.

Moher, D. (2009) 'Liberati A, Tetzlaff J, Altman DG, Group TP', *Preferred reporting items for systematic reviews and meta-analyses: the PRISMA statement.PLoS Med,* 6(7), pp. 1000097.

Navickas, R., Petric, V., Feigl, A.B. and Seychell, M. (2016) 'Multimorbidity: what do we know? What should we do?', *Journal of comorbidity,* 6(1), pp. 4-11.

O'Brien, R., Wyke, S., Guthrie, B., Watt, G. and Mercer, S. (2011) 'An ‘endless struggle’: a qualitative study of general practitioners’ and practice nurses’ experiences of managing multimorbidity in socio-economically deprived areas of Scotland', *Chronic illness,* 7(1), pp. 45-59.

Ouzzani, M., Hammady, H., Fedorowicz, Z. and Elmagarmid, A. (2016) 'Rayyan—a web and mobile app for systematic reviews', *Systematic reviews,* 5(1), pp. 1-10.

Palmer, K., Marengoni, A., Forjaz, M.J., Jureviciene, E., Laatikainen, T., Mammarella, F., Muth, C., Navickas, R., Prados-Torres, A. and Rijken, M. (2018) 'Multimorbidity care model: Recommendations from the consensus meeting of the Joint Action on Chronic Diseases and Promoting Healthy Ageing across the Life Cycle (JA-CHRODIS)', *Health Policy,* 122(1), pp. 4-11.

Pillay-van Wyk, V., Gbabe, O.F., Roomaney, R., Nglazi, M., Joubert, J. and Bradshaw, D. (2015) *Risk of bias tool for systematic review of observational studies.* pp. 1.

Shamseer, L., Moher, D., Clarke, M., Ghersi, D. and Liberati, A. (2015) 'Research methods & reporting', *BMJ,* , pp. 1-25.

Sum, G., Salisbury, C., Koh, G.C., Atun, R., Oldenburg, B., McPake, B., Vellakkal, S. and Lee, J.T. (2019) 'Implications of multimorbidity patterns on health care utilisation and quality of life in middle-income countries: cross-sectional analysis', *Journal of global health,* 9(2).

Von Elm, E., Altman, D.G., Egger, M., Pocock, S.J., Gøtzsche, P.C. and Vandenbroucke, J.P. (2007) 'The Strengthening the Reporting of Observational Studies in Epidemiology (STROBE) statement: guidelines for reporting observational studies', *Bulletin of the World Health Organization,* 85, pp. 867-872.

Xu, X., Mishra, G.D. and Jones, M. (2017) 'Mapping the global research landscape and knowledge gaps on multimorbidity: a bibliometric study', *Journal of global health,* 7(1).

Yoon, J., Zulman, D., Scott, J.Y. and Maciejewski, M.L. (2014) 'Costs associated with multimorbidity among VA patients', *Medical care,* , pp. S31-S36.
