# Supplementary material for: Systematic Literature Review of the Prevalence, Pattern, and Determinant of Multimorbidity Among Older Adults in Nigeria
Source: Health Serv Res Manag Epidemiol. 2023 Jun 26;10:23333928231178774. doi: 10.1177/23333928231178774 (PMC10331101; doi:10.1177/23333928231178774)
Supplement: sj-docx-3-hme-10.1177_23333928231178774 - Supplemental material for Systematic Literature Review of the Prevalence, Pattern, and Determinant of Multimorbidity Among Older Adults in Nigeria [file sj-docx-3-hme-10.1177_23333928231178774.docx]

|  | Nwani and Isah, 2016 (Anambra state) | Adams and Abubakar, 2018 (Abuja) | Olawumi *et al.,* 2021 (Kano) | Abdulraheem et al., 2017 (Niger) | Faronbi, Ajadi and Gobbens, 2020 (Osun state) | Abdulazeez *et al.,* 2021 (Kano) |
| --- | --- | --- | --- | --- | --- | --- |
| Was the sample frame appropriate to address the target population? | YES | YES | YES | YES | YES | YES |
|  |  |  |  |  |  |  |
| Were study participants sampled in an appropriate way? | UNCLEAR | NOT APPLICABLE | YES | YES | YES | YES |
|  |  |  |  |  |  |  |
| Was the sample size adequate? | NOT APPLICABLE | NOT APPLICABLE | YES | YES | YES | YES |
|  |  |  |  |  |  |  |
| Were the study subjects and the setting described in detail? | NO | YES | YES | NO | YES | YES |
|  |  |  |  |  |  |  |
| Was the data analysis conducted with sufficient coverage of the identified sample? | UNCLEAR | YES | YES | YES | YES | YES |
|  |  |  |  |  |  |  |
| Were valid methods used for the identification of the condition? | YES | YES | YES | YES | YES | YES |
|  |  |  |  |  |  |  |
| Was the condition measured in a standard, reliable way for all participants? | YES | YES | YES | YES | YES | YES |
|  |  |  |  |  |  |  |
|  |  |  |  |  |  |  |
| Was there appropriate statistical analysis? | YES | YES | YES | YES | YES | YES |
|  |  |  |  |  |  |  |
| 9. Was the response rate adequate, and if not, was the low response rate managed appropriately? | YES | YES | YES | YES | YES | YES |
|  |  |  |  |  |  |  |
| Overall appraisal: | 62.5 | 100% | 100% | 88% | 100% | 100% |
|  |  |  |  |  |  |  |
