# Supplementary material for: Systematic Literature Review of the Prevalence, Pattern, and Determinant of Multimorbidity Among Older Adults in Nigeria
Source: Health Serv Res Manag Epidemiol. 2023 Jun 26;10:23333928231178774. doi: 10.1177/23333928231178774 (PMC10331101; doi:10.1177/23333928231178774)
Supplement: sj-docx-5-hme-10.1177_23333928231178774 - Supplemental material for Systematic Literature Review of the Prevalence, Pattern, and Determinant of Multimorbidity Among Older Adults in Nigeria [file sj-docx-5-hme-10.1177_23333928231178774.docx]

1 Table: Articles screened for full text

| S/N | Author | Title | Decision | Reason |
| --- | --- | --- | --- | --- |
| 1 | Nwani and Isah, 2016 (Anambra state) | Chronic diseases and multimorbidity among elderly patients admitted in the medical wards of a Nigerian tertiary hospital | Included | Meets eligibility criteria |
| 2 | Adams and Abubakar, 2018 (Abuja) | Morbidity Patterns of Elderly Patients Attending the General Out-Patient Clinic of a Tertiary Centre in North-Central – Nigeria | Included | Meets eligibility criteria |
| 3 | Osunkwo *et al.,* 2018 | Mortality Pattern at the National Hospital: A Hospital-Based Study in Abuja, Nigeria | Excluded | Wrong study design |
| 4 | Olawumi *et al.,* 2021 (Kano) | Nutritional Status and Morbidity Patterns of the Elderly in a North-western Nigerian Hospital: A Cross-sectional Study | Included | Meets eligibility criteria |
| 5 | Abdulraheem et al., 2017 (Niger) | Prevalence and Pattern of Multi-Morbidity among Elderly People in Rural Nigeria: Implications for Health Care System, Research and Medical Education | Included | Meets eligibility criteria |
| 6 | Nguyen *et al.,* 2019 | Prevalence of multimorbidity in community settings: A systematic review and meta-analysis of observational studies | Excluded | Wrong study design |
| 7 | Disang, Weller and Campbell, 2021 | Prevalence and patterns of chronic communicable and noncommunicable diseases multimorbidity in sub-Saharan Africa: protocol for a systematic review | Excluded | Wrong study design |
| 8 | Faronbi, Ajadi and Gobbens, 2020 (Osun state) | Associations of chronic illnesses and socio-demographic factors with health-related quality of life of older adults in Nigeria: A cross-sectional stud | Included | Meets eligibility criteria |
| 9 | Agofure, Okandeji-Barry and Ogbon, 2020 | Pattern of Diabetes Mellitus Complications and Co‑morbidities in Ughelli North Local Government Area, Delta State, Nigeria | Excluded | Wrong study outcome |
| 10 | Akpa *et al.,* 2013 | Profile and Outcome of Medical Emergencies in a Tertiary Health Institution in Port Harcourt, Nigeria | Excluded | Wrong study outcome |
| 11 | Salako *et al.,* 2018 | The pattern of comorbidities in cancer patients in Lagos, South-Western Nigeria | Excluded | Wrong study outcome |
| 12 | Amedu and Sale, 2020 | Prevalence of psychiatric disorders among elderly in-patients in non-psychiatric wards of a teaching hospital in Northern Nigeria | Excluded | Wrong study design |
| 13 | Abdulazeez *et al.,* 2021 (Kano) | Multimorbidity and Functional Status of the Elderly in a Primary Care Setting of Northern Nigeria: A Cross-Sectional Stud | Included | Meets eligibility criteria |
| 14 | Cadmus *et al.,* 2017 | A descriptive study of the morbidity pattern of older persons presenting at a Geriatric Centre in Southwestern Nigeria', *Nigerian journal of clinical practice,* 20(7), pp. 873-878. | Excluded | Wrong study outcome |
